# Self-Powered Long-Life Microsystem for Vibration Sensing and Target Recognition

**DOI:** 10.3390/s22249594

**Published:** 2022-12-07

**Authors:** Deng Yang, Wenrui Duan, Guozhe Xuan, Lulu Hou, Zhen Zhang, Mingxue Song, Jiahao Zhao

**Affiliations:** 1 Department of Precision Instrument, Tsinghua University, Beijing 100084, China; 2 Key Laboratory of Smart Microsystem (Tsinghua University) Ministry of Education, Tsinghua University, Beijing 100084, China; 3 State Key Laboratory of Precision Measurement Technology and Instruments, Tsinghua University, Beijing 100084, China; 4 Beijing Laboratory of Biomedical Detection Technology and Instrument, Beijing 100084, China; 5 Beijing Advanced Innovation Center for Integrated Circuits, Beijing 100084, China; 6 School of Instrument Science and Opto-Electronics Engineering, Beijing Information Science and Technology University, Beijing 100192, China

**Keywords:** microsystem, long-life, self-powered, vibration sensing, target recognition, low-power consumption, support vector machine

## Abstract

Microsystems play an important role in the Internet of Things (IoT). In many unattended IoT applications, microsystems with small size, lightweight, and long life are urgently needed to achieve covert, large-scale, and long-term distribution for target detection and recognition. This paper presents for the first time a low-power, long-life microsystem that integrates self-power supply, event wake-up, continuous vibration sensing, and target recognition. The microsystem is mainly used for unattended long-term target perception and recognition. A composite energy source of solar energy and battery is designed to achieve self-powering. The microsystem’s sensing module, circuit module, signal processing module, and transceiver module are optimized to further realize the small size and low-power consumption. A low-computational recognition algorithm based on support vector machine learning is designed and ported into the microsystem. Taking the pedestrian, wheeled vehicle, and tracked vehicle as targets, the proposed microsystem of 15 cm^3^ and 35 g successfully realizes target recognitions both indoors and outdoors with an accuracy rate of over 84% and 65%, respectively. Self-powering of the microsystem is up to 22.7 mW under the midday sunlight, and 11 min self-powering can maintain 24 h operation of the microsystem in sleep mode.

## 1. Introduction

With the development of the Internet of Things (IoT), microsystems, which are an important part of the IoT, have attracted more and more attention [1]. In many unattended scenarios, microsystems are used for target detection and recognition by sensing signals such as vibration, sound, RF, infrared, magnetic, and pressure signal [2,3]. The vibration signal is one of the commonly used signals for target recognition [4]. Since different targets produce vibrations with different characteristics, and vibration signals can travel a long distance along the ground, target recognition based on the vibration signal is a feasible solution for microsystems [5].

Microsystems are often powered by batteries with limited battery life, which contradicts the requirement for long-term, large-scale distribution [6]. Low-power consumption [7] and self-powered technologies [8,9], especially based on vibration [10,11,12], are studied to improve the microsystem endurance. The use of complex self-powered energy modules and high-performance processors increases the size and power consumption of microsystems, while the use of low-power components reduces system performance. The microsystem with limited performance, such as low-precision sensing and low-computing power, hinders its signal sensing and target recognition capabilities [13]. In many cases, data acquired from microsystem sensors are sent to the parent system for further processing, which puts a burden on the parent system [14,15,16]. This creates problems such as high latency and high communication power consumption. With the rapid increase in the number of terminals in IoT, autonomous perception and recognition by microsystems is becoming more and more important [17]. Therefore, balancing between self-power supply, low-power consumption, sensing, and recognition capabilities is a system optimization problem worth studying and practicing.

Some microsystems for vibration sensing have been reported, as shown in Table 1. A system for vehicle detection is proposed [18]. However, the appearance, compositions, and related parameters of the system have not been clarified. Then a self-powered microsystem for vibration sensing and transmission is reported [19]. Since the charging power is lower than the system power consumption, the system can only work periodically instead of continuously. A self-powered system with an event wake-up function is studied [20]. The charging power is only 64 μW, it takes 21 s of charging to send a set of data, and the modules of the system have not been well integrated. Another vibration sensing and transmitting system has been reported recently [21]. The charging power is increased to almost 1 mW, and data can be transmitted after charging for 0.64 s. However, the system has a volume of up to 300 cm^3^ and a weight of up to 1 kg. A self-powered microsystem with both event wake-up function and target recognition ability is investigated, and the system volume is about 144 cm^3^ [22]. However, vigorous shaking for 4 to 5 s is required for collecting enough energy to wake up the system, and the recognition is simply based on threshold and frequency. These energy-harvester-based microsystems take several seconds to power on, which means that the vibration sensing of the microsystems is not continuous. Till now, microsystems for continuous vibration sensing with small size, self-powered, event wake-up, and machine-learning-based recognition have not been reported.

**Figure 1:**
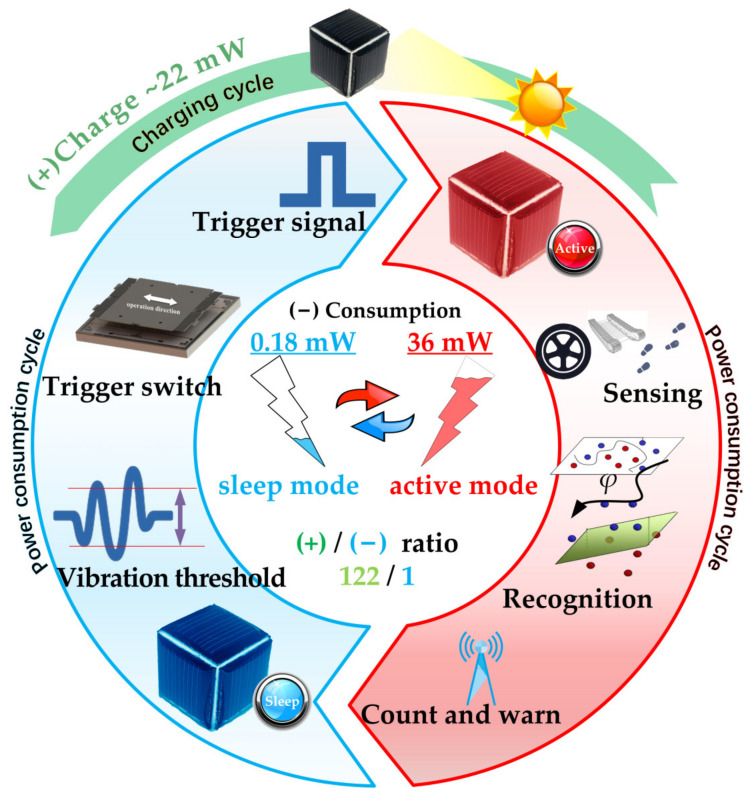
Overall working principle of microsystem.

In this paper, a self-powered microsystem with event wake-up, vibration sensing, and target recognition capabilities is designed and implemented for the first time. Hardware modules and software algorithms of the microsystem are optimized as a whole to reduce volume, weight, and power consumption, as well as to enhance online target recognition. The overall working principle of the microsystem is shown in Figure 1. An energy module composed of a lithium battery and photovoltaic panels is designed for self-powering. A MEMS accelerometer in the microsystem is used for vibration sensing, and a vibration trigger switch is used to realize the wake-up of the microsystem. A support vector machine (SVM)-based recognition algorithm is designed and ported into the microsystem’s MCU for target recognition. The microsystem’s performances, including self-powering, power consumption, vibration sensing, and target recognition, are verified by tests and experiments. As an IoT terminal, this microsystem can be effectively used in numerous unattended applications.

## 2. Microsystem Design and Implementation

In unattended applications, low-power consumption and self-powering are the keys to long-life microsystems. In this paper, modules of the microsystem are optimally designed to realize vibration perception and recognition, while maintaining the characteristics of small size, low-power consumption, and self-supply of the microsystem.

**Figure 2:**
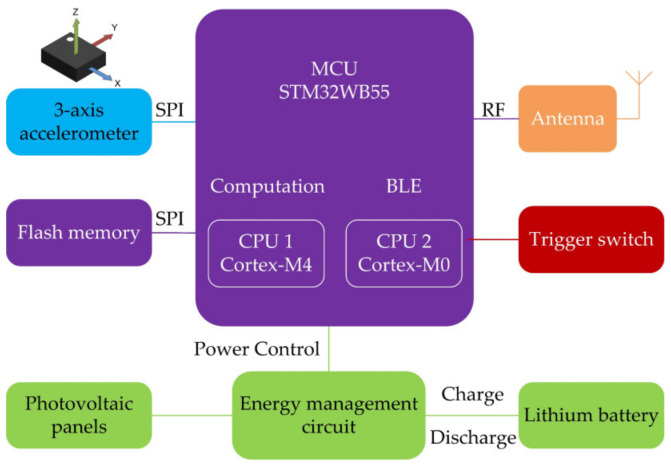
Microsystem block diagram.

The composition of the microsystem is shown in Figure 2. The selected main MCU is a low-power processor with a Bluetooth chip included, which is used for data processing and transmission. A MEMS tri-axis accelerometer is applied for vibration sensing, and it sends vibration data to the MCU through the SPI interface. A MEMS vibration trigger switch is used to sense the presence of targets and trigger the microsystem. An antenna is integrated for RF communication. A power module, composed of photovoltaic panels, a lithium battery, and a supporting energy management circuit, is used for the system power supply.

### 2.1. Main Board Module

**Figure 3:**
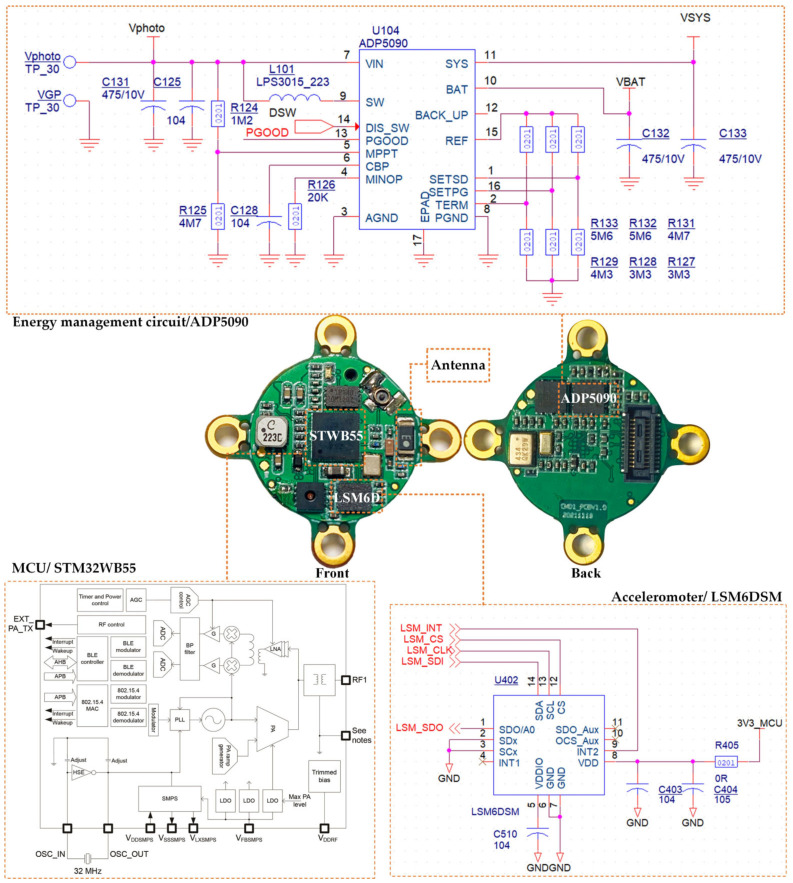
Main board of microsystem.

The MCU, tri-axis accelerometer, antenna, and energy management circuit are compactly integrated into a single PCB, named the main board, to reduce the size of the microsystem, as shown in Figure 3.

The MCU, model STM32WB55, designed for ultra-low-power consumption, has a floating-point unit (FPU) that supports single-precision data processing, and an embedded memory (1 MB Flash, 256 k SRAM) that meets the computing and data processing required in this system. In addition, the MCU supports multiple operating modes, including sleeping mode and active mode, which is the basis of event wake-up.

The tri-axis accelerometer, model LSM6DSM, is also an ultra-low-power MEMS device that draws only 0.4 mA in normal mode and 9 μA at a 12.5 Hz sampling rate.

The energy management circuit is responsible for the energy management of charging and discharging. Photovoltaic panels collect light energy and convert it into electrical energy, but the low-voltage current cannot charge the battery directly. An ADP5090 chip integrates a nanometer power boost regulator, and an energy storage element management controller is applied in the circuit. It converts the output power of low-voltage, high-impedance DC sources from the PVs. The circuit stores electrical energy in a rechargeable battery with energy storage protection and provides power to the load. The circuit also has over-discharge protection and overcharge protection to make the microsystem safer and more reliable.

### 2.2. Self-Power Module

**Figure 4:**
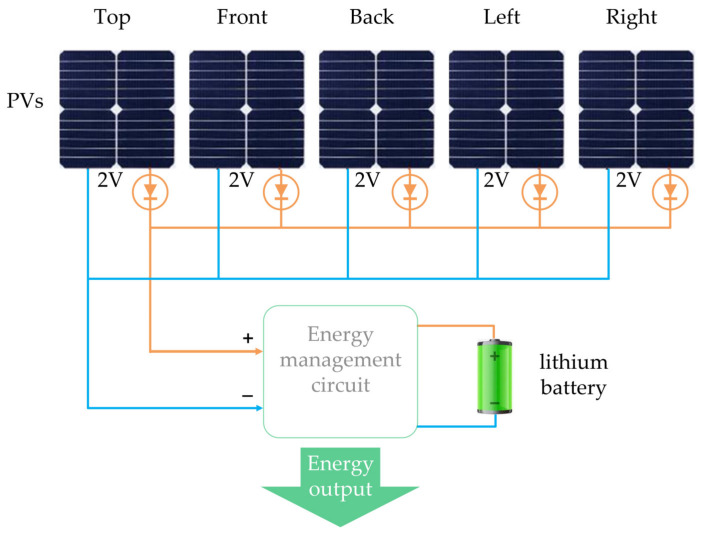
Self-powered module of microsystem.

The self-powered module is used for energy harvesting and storage, which is composed of five monocrystalline silicon photovoltaic panels (PVs) of 20 mm × 20 mm in parallel and a Φ16 mm × 5.5 mm lithium battery, as shown in Figure 4. A single no-load voltage of the PVs is about 2 volts. To avoid mutual interference between the parallel PVs, which will affect the overall self-powering efficiency, five diodes are adopted to ensure the overall conversion efficiency. The circuit of self-powered module is presented in Figure 4.

### 2.3. Vibration Trigger Switch

The MEMS vibration trigger [23] switch is used to filter out most of the non-target vibrations in unattended scenarios. The threshold of the switch can be as low as 0.6 g, and the operational power consumption has been tested to be from 1.32 to 1.50 μW.

### 2.4. Microsystem Assembly

**Figure 5:**
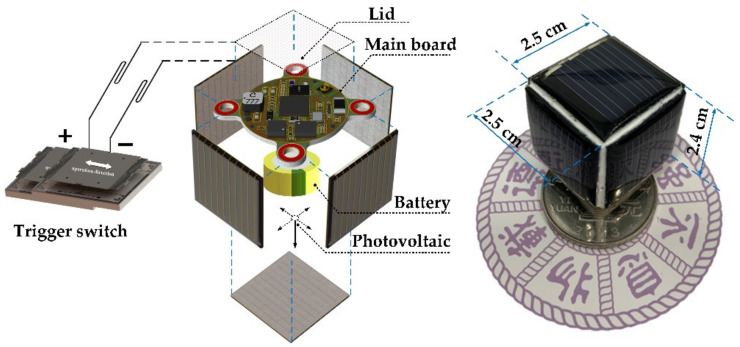
Structure and components of the microsystem.

The microsystem hardware integration is shown in Figure 5. The main board, five photovoltaic panels, lithium battery, and vibration trigger switch are assembled firmly into a 3D printed hexahedron frame, which ensures the positions and shock resistances of the microsystem modules. The five PVs are located on different sides of the frame to ensure effective charging under different attitudes of the microsystem. The microsystem is cubic with side lengths of 2.5 cm, 2.5 cm, and 2.4 cm, whose volume is 15 cm^3^ and weight is 35 g.

## 3. Recognition Algorithm and Software Design

**Figure 6:**
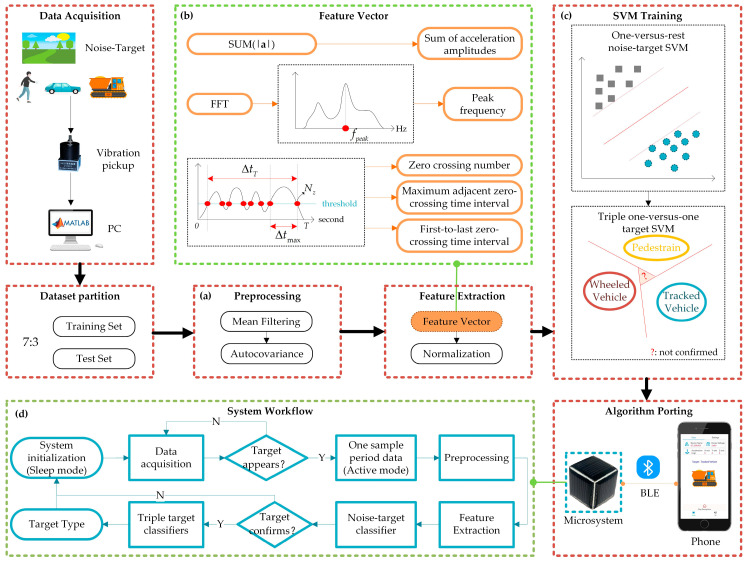
Recognition Algorithm and Software Design.

A recognition algorithm and software flow are designed for target recognition, as shown in Figure 6. Pedestrians, wheeled vehicles, and tracked vehicles are potential monitoring objectives, which are also used as targets in this paper. Limited by the low-accuracy MEMS vibration sensor and the low-computing-power MCU in the microsystem, a simple and efficient recognition algorithm is required. Preprocessing of the data is applied to improve the signal-to-noise ratio of the original data first. After that, a low-computation SVM classification algorithm is used for target recognition. To further reduce the microsystem power consumption, the microsystem is designed to work in two states—low-power sleep mode and high-power active mode.

### 3.1. Preprocessing

The movement of pedestrians, wheeled vehicles, and crawler vehicles on the ground causes the deformation of the non-rigid earth medium, and the deformation propagates in the earth to generate vibration signals. During this process, due to the interference of the medium propagating properties, sensor errors, and other vibration source disturbances, the signal-to-noise ratio of the target vibration signal decreases rapidly with the increase in the distance between the sensor and the target, which affects the detection and recognition of the target. Selecting appropriate methods to preprocess the original data can improve the signal quality significantly [13]. In this paper, the mean filtering and autocovariance method are used for preprocessing with small computational complexity, as shown in Figure 6a. The mean filter effectively removes the DC noise in the signal [24], and the autocovariance method extracts the target periodic signal submerged in the random noise [25].

### 3.2. Feature Extraction

Signal features need to be extracted before applying the SVM classification. The greater the difference in the signal features of the target, the better the performance of the classification. Considering the characteristics of the different targets’ vibration signal in the time domain and frequency domain, the following five features are extracted—sum of acceleration amplitudes *a_sum_*, peak frequency *f_peak_*, zero-crossing number *N_z_*, maximum adjacent zero-crossing time interval Δ*t_max_*, and first-to-last zero-crossing time interval Δ*t_T_*, as shown in Figure 6b. All five features are discussed within one signal sampling period defined in the software flow below. These features have the advantages of low-computational complexity and easy hardware implementation in microsystems [26].

Sum of acceleration amplitudes *a_sum_*—Acceleration amplitude reflects the energy of the vibration. The force on the ground during the movement of different targets is very different, so the vibration energy they generate is very different [27,28]. *a_sum_* is calculated by summing the acceleration amplitudes within a sampling period.

Peak frequency *f_peak_*—The peak frequency reflects the main frequency components of the vibration signal. The main frequencies of people and vehicles are significantly different [29] The spectral distribution is obtained by performing a fast Fourier transform (FFT) on the vibration signal, and the frequency corresponding to the peak spectral density is found as the peak frequency.

Zero-crossing features—Zero-crossing features refer to comparing the time domain signal amplitude with a set threshold and calculating the number and frequency of the positive or negative crossing of the threshold. Zero-crossing features reflect the characteristics of the signal in both amplitude and frequency, and they are easy to be extracted. The zero-crossing features of the vibration signals generated by pedestrians and vehicles during moving are distinguishable [30]. Thus, three zero-crossing-related features are selected as the signal features in this paper, which are the zero-crossing number *N_z_*, the maximum adjacent zero-crossing time interval Δ*t_max_*, and the first-to-last zero-crossing time interval Δ*t_T_*, respectively.

To sum up, the five features of the vibration signal are extracted for the SVM classification, and mean-standard deviation normalization is performed for each feature to improve the classification performance. The formula is as follows:

(1) $$\left\{\begin{array}{l} a_{sum}=\sum_{i=1}^{N}\left|a(i)\right|\\ f_{peak}=f:\max\left(S_{f}\right)\\ N_{z}=\frac{1}{2}\sum_{i=2}^{N}\left|\mathrm{sgn}[a_{z}(i)]-\mathrm{sgn}[a_{z}(i-1)]\right|\\ \Delta t_{\max}=\max\left[t\left(j\right)-t\left(j-1\right)\right]\\ \Delta t_{T}=t\left(N_{z}\right)-t\left(1\right) \end{array}\right.$$

where **a**(*i*) is the *i*-th tri-axis acceleration vector, and *t*(*j*) is the time of the *j*-th zero-crossing point.

### 3.3. SVM Training

There are many kinds of machine learning algorithms and models that can identify vibration signals in unattended areas. In this paper, based on the hardware composition of the microsystem, an algorithm with small computation and memory is required. The comparison of signal operation computation is shown in Table S1. Considering the power consumption and low-computation performance of the microsystem, an SVM algorithm with a small amount of training and computation is designed, which makes a compromise in the recognition accuracy.

SVM is a data classification method based on statistical learning. Its principle is to divide the samples belonging to different categories into both sides of a hyperplane in the feature space. It is often used to solve binary classification problems. At present, there are two kinds of multi-classification methods based on SVM, which are one-versus-one and one-versus-rest [31]. The one-versus-one method refers to the construction of an SVM classifier between every two classes during training, and the multiple classifiers vote during testing, taking the one with the most votes as the final target category. This method avoids the problems such as the reduction in classification accuracy caused by the imbalance of positive and negative samples and the need to retrain classifiers when adding new categories [32].

In this paper, a one-versus-rest noise-target classifier is applied first to classify the targets from the environmental random vibrations. After that, triple one-to-one target classifiers are used to achieve the classification of the pedestrian, wheeled vehicle, and tracked vehicle, as shown in Figure 6c. The main code of SVM is shown in Table S2. If there is an equal number of votes after the classifiers, the target category cannot be confirmed. In this paper, the microsystem is mainly used for hazard counting and alarming that a small number of unclassified alarms are allowed. Improving the classification performance requires more complex calculations at the cost of higher power consumption. This paper selects a design that compromises performance and power consumption.

### 3.4. System Workflow

Considering the limited hardware resources of the microsystem, especially the energy, and the target recognition performance, the software workflow of the microsystem is optimized, including system initialization, target initial detection, system working mode switching, preprocessing, feature extraction, target re-detection, and final target classification, as shown in Figure 6d. In the system initialization, determine the signal sampling period, accelerometer sampling rate, and ambient noise threshold, and set the system into low-power sleep mode. In sleep mode, the accelerometer for vibration sensing remains on, while the rest of the microsystem remains in a low-power state. In the target initial detection, whether a target appears or not is judged based on the acceleration amplitude. If the sum of the acceleration amplitudes in one sample period is greater than five times the set ambient noise threshold, or greater than two times the sum value of the previous sample period, it is determined that a target appears, and the microsystem switches to high-power active mode. The one-versus-rest classifier above is applied for the target re-detection, and triple one-to-one classifiers are used for the final target classification.

## 4. Results and Discussion

### 4.1. Power Consumption and Self-Powering

Power consumption in low-power sleep mode and high-power active mode is tested, respectively. The power consumption is 0.18 mW in sleep mode, while the power consumption increases to 36 mW in active mode.

**Figure 7:**
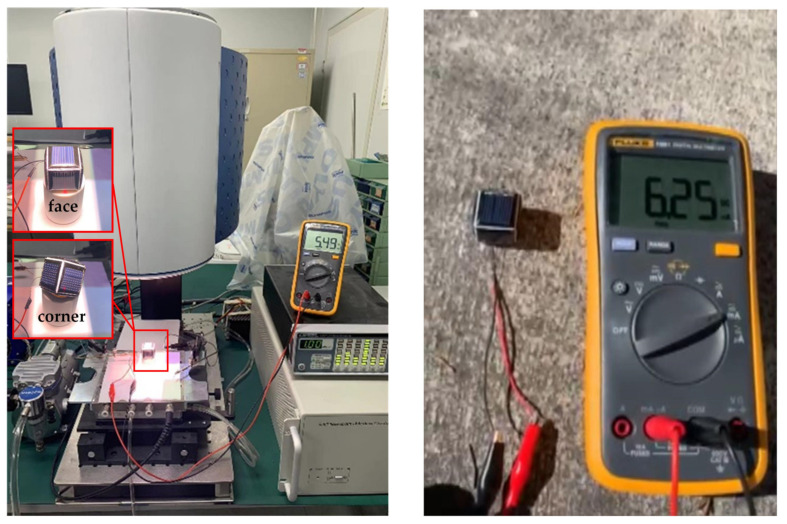
Self-power performance testing under simulated and natural sunlight.

The self-powered performance of the microsystem is verified under both the solar simulator and natural light conditions, as shown in Figure 7. Under the indoor condition, a solar simulator (DAIEL LSS-7120) with an output intensity of 1 kW/m^2^ is used to irradiate the microsystem at a distance of 200 mm. Under outdoor condition, the microsystem is irradiated by the midday sunlight on a sunny day. The self-powered performance at different incident angles is tested to simulate the incident conditions at different times of a day.

The power consumption and self-powering results are shown in Table 2. The self-powering of the microsystem in different attitudes is similar, which verifies the charging efficiency and stability during the actual application process. The self-powering under midday sunlight is about 126 times the power consumption in sleep mode, which means that 11 min of self-powering maintain 24 h microsystem operation in sleep mode. Thus, the long life of the microsystem is achieved.

### 4.2. Sensing and Recognition Experiment

**Figure 8:**
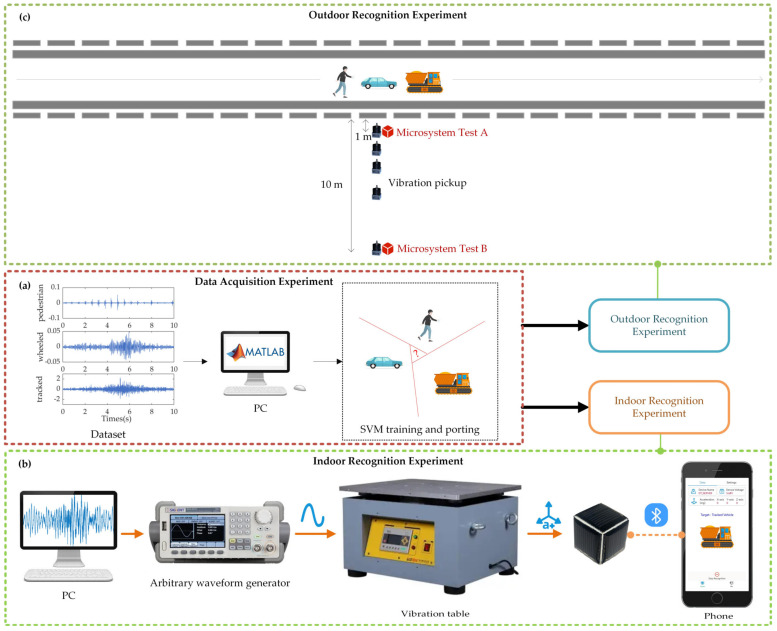
Sensing and Recognition Experiment.

In this experiment, a pedestrian, a tracked vehicle, and a wheeled vehicle are used as the recognition targets, as shown in Figure 8. Firstly, one of the targets travels along the test path at a constant speed, and vibration data of the target are collected by five high-accuracy vibration sensors. The data are then used to train the recognition algorithm. After that, the trained recognition algorithm is ported into the microsystem. Finally, the microsystem is applied for target recognition both indoors and outdoors.

#### 4.2.1. Data Acquisition Experiment

The setup of the data acquisition experiment is shown in Figure 8a. The vibration data of the pedestrian, wheeled vehicle, and tracked vehicle are collected on a flat soil pavement of 150 m long and 5 m wide. The five high-accuracy vibration sensors are placed at the midpoint of the pavement and 1, 2, 3, 5, and 10 m away from the edge of the pavement. The targets move along the centerline of the pavement at a given speed, and the parameters are shown in Table 3.

**Figure 9:**
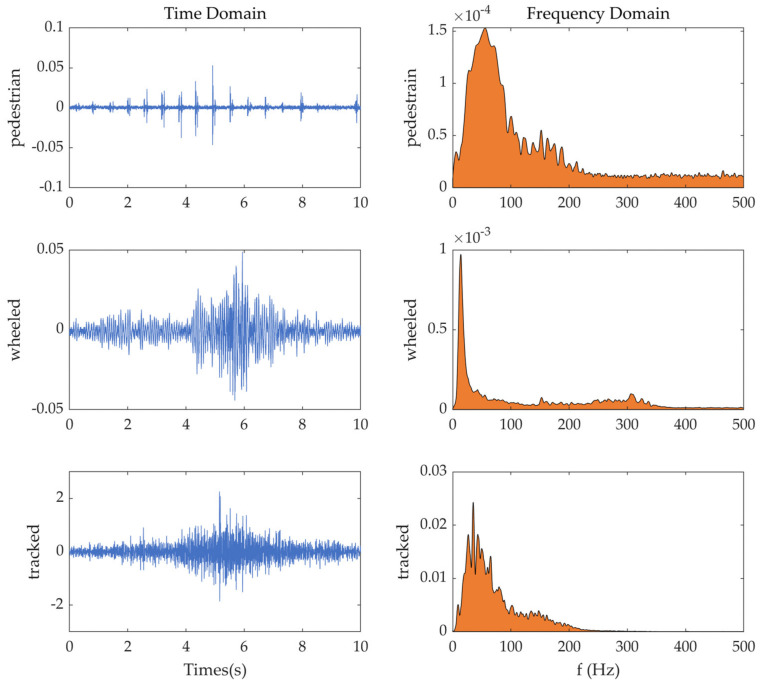
Targets’ Vibration Signal Distribution in time domain and frequency domain.

Choosing one group from the data groups as an example, the distribution of the targets’ vibration signal in the time domain and frequency domain is shown in Figure 9. In the time domain, the vibration amplitude of the pedestrian is the smallest, followed by the wheeled vehicle, and the largest by the tracked vehicle. In addition, the pedestrian’s vibration signal is characterized by periodic pulses. In the frequency domain, the frequency distribution of the pedestrian is the widest, followed by the tracked vehicle and the narrowest of the wheeled vehicles. The peak frequencies of the pedestrian, wheeled vehicle, and tracked vehicle are about 55 Hz, 15 Hz, and 35 Hz, respectively.

In the collected data, 60 groups of the tracked vehicle, 50 groups of the wheeled vehicle, 60 groups of the pedestrian, and 50 groups of environmental signals are selected for SVM training. The collected data are divided into a training set and a test set with a ratio of 7:3. After training, the SVM classifiers for target recognition are obtained and ported into the microsystem for the following indoor and outdoor recognition.

#### 4.2.2. Indoor Recognition Experiment

There are many uncontrollable factors in outdoor experiments, and the cost is high. To ensure the quality and efficiency of the recognition experiment, this paper builds an indoor experimental platform firstly to simulate the actual ground vibration based on the acquired vibration data above. The setup for the indoor experiment is shown in Figure 8b, and a more detailed setup is shown in Figure S1. The vibration data collected by the high-accuracy vibration sensors are loaded into a PC and are used to control a vibration table to simulate the real vibration inputs. The microsystem is assembled on the vibration table for vibration sensing and target recognition.

The indoor recognition results are shown in Table 4. The recognition accuracies of all the three targets are higher than 84%. Thus, the target recognition ability of the microsystem is verified.

#### 4.2.3. Outdoor Recognition Experiment

**Figure 10:**
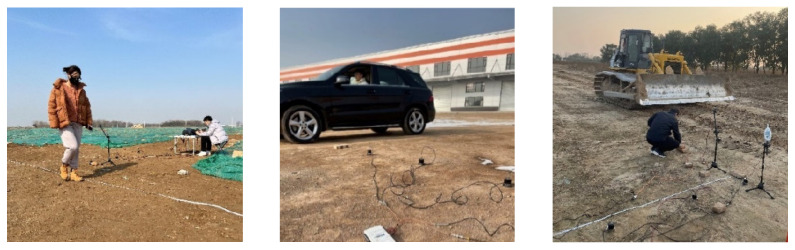
Outdoor experiment setup.

The setup for the outdoor experiment is shown in Figure 10, which is similar to the setup in the data acquisition experiment, except that the high-precision vibration sensors are replaced by the microsystem. In test A, the microsystem is placed 1 m away from the edge of the pavement, and the wheeled vehicle and the pedestrian are applied for recognition. In test B, the microsystem is placed 10 m away and the tracked vehicle is applied for recognition.

The outdoor recognition results are shown in Table 5. The recognition accuracies of all the three targets are higher than 65%.

Comparing the experimental results of the indoor and outdoor experiments, the recognition accuracy of various targets in the wild is lower than indoors. Fortunately, the tracked vehicle is much easier to distinguish than the pedestrian and the wheeled vehicle. The main reasons for these results are: (a) In the indoor experiment, vibrations are effectively applied to the accelerometer in the microsystem through the vibration table. However, in the outdoor experiment, due to the large difference in the compactness of the soil road surface and the contact interface between the microsystem and the ground, the vibration cannot effectively act on the accelerometer, resulting in poor quality of the vibration signal sensed by the accelerometer; (b) Compared with the other two targets, the vibration signal quality of the tracked vehicle is less degraded because its vibration energy is larger and less susceptible to interference.

**Figure 11:**
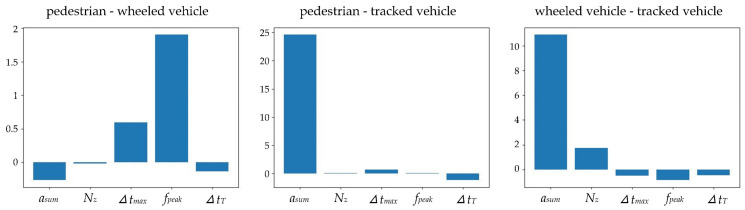
Feature importance of SVM with linear kernel coefficient.

There are several ways to further improve the recognition accuracy:

(1) Increasing the microsystem’s weight or burying the microsystem in the soil. These methods can increase the contact force between the microsystem and the ground, so that the vibration signal can better act on the accelerometer, thereby improving the quality of the vibration signal. However, these methods are contrary to the microsystem advantages of small size, lightweight, and easy deployment.

(2) Collecting more samples for training the machine learning model. The performance of recognition algorithms based on machine learning largely depends on training samples; however, this places higher demands on the memory size and computing power of the on-site microsystem.

(3) Selecting features with more efficiencies. The greater the difference in features between different targets, the better the features are for recognition. Finding a way to point out key features is meaningful. In this paper, an SVM “poly” kernel coefficient is used for target recognition after plenty of experiments on different kernel coefficients. Thus, the SVM is a non-linear model. It is difficult for nonlinear SVM to explicitly point out the feature importance. The reason is that when the SVM is nonlinear, the dataset is mapped to a higher dimensional space, which makes it difficult to find the element importance related to the parent dataset element. Therefore, an SVM with a linear kernel coefficient is used to rank the importance of the features indirectly. The ranking results are shown in Figure 11. It can be seen from the figure that *f_peak_* is the most important feature to distinguish the pedestrian from the wheeled vehicle, and *a_sum_* is the most important feature to distinguish the tracked vehicle from the other two targets. In this way, feature selections for recognition algorithms can be optimized in future works.

## 5. Conclusions

In this paper, a low-power, long-life microsystem with the capabilities of self-power supply, event wake-up, continuous vibration sensing, and target recognition is designed and implemented for the first time. The hardware modules are optimized to ensure the small size, lightweight, and low-power consumption of the microsystem. A composite energy source composed of a lithium battery and five photovoltaic panels is designed to achieve self-powering. Considering the limited hardware conditions and low-power consumption requirements of the microsystem, a sufficient recognition algorithm based on SVM is designed and a low-power software workflow with a wake-up function is designed and ported into the microsystem. The microsystem of 15 cm^3^ and 35 g is implemented. The power consumption of the microsystem in sleep mode and active mode is 0.18 mW and 36 mW, respectively, while the self-powering of the microsystem is up to 22.7 mW under the midday sunlight. Self-powering for 11 min maintains the microsystem working in sleep mode for 24 h. Indoor and outdoor experiments for the pedestrian, wheeled vehicle, and tracked vehicle recognition are carried out and the recognition accuracy rate is higher than 84% and 65%, respectively. Event wake-up, self-power supply, and self-recognition are crucial to constructing an unattended, long-life, wide-area distributed monitoring microsystem network. Further improving system performances based on this work, such as smaller size, lower power consumption, more robust self-power supply, and better recognition capabilities, will promote the development of unattended surveillance networks.

## Figures and Tables

**Table 1:** Vibration sensing microsystems.

Refs	Size	Self- Powered (+)	Power Consumption (−)	(+)/(−) Ratio	Event Wake-Up	Continuous Sensing	Target Recognition	Year
[18]	-	-	-	-	-	Yes	wheeled vehicle tracked vehicle	2004
[19]	-	45 μW	109 μW	0.41	No	No	No	2007
[20]	-	64 μW	1.4 mJ per transmission	21 s *	Yes	No	No	2015
[21]	9 × 6.8 × 5 cm^3^	<1 mW	0.64 mJ per transmission	0.64 s *	Yes	No	No	2022
[22]	6 × 6 × 4 cm^3^	0.74 μW	-	4–5 s *	Yes	No	hand-shake hammer-knock	2018
this study	2.5 × 2.5 × 2.4 cm^3^	22.7 mW	0.18 mW	126	Yes	Yes	pedestrian wheeled vehicle tracked vehicle	-

* Charging time per transmission.

**Table 2:** Self-power performance testing (mW).

Power Consumption		Self-Powering			
Sleep Mode	Active Mode	Solar Simulator		Natural Light	
		Face	Corner	Face	Corner
0.18	35.88	19.76	24.62	20.23	22.72

**Table 3:** Target movement parameters.

Target	Weight (kg)	Speed (km/h)
pedestrian	65, 65, 70	4–8
wheeled vehicle	2135	10–30
tracked vehicle	18,000	3–11

**Table 4:** Indoor recognition results.

Target	Sample Number	Correct Number	Accuracy (%)
tracked vehicle	106	98	92.4
wheeled vehicle	25	21	84.0
pedestrian	30	26	86.7

**Table 5:** Outdoor recognition results.

Target	Sample Number	Correct Number	Accuracy (%)
tracked vehicle	50	45	90.0
wheeled vehicle	20	13	65.0
pedestrian	40	26	65.0

## Supplementary Materials

The following supporting information can be downloaded at: https://www.mdpi.com/article/10.3390/s22249594/s1, Figure S1: Indoor experiment setup; Table S1: Comparison of signal operation computation; Table S2: SVM code.
