# Supplementary material for: Self-Powered Long-Life Microsystem for Vibration Sensing and Target Recognition
Source: Sensors (Basel). 2022 Dec 7;22(24):9594. doi: 10.3390/s22249594 (PMC9783103; doi:10.3390/s22249594)
Supplement: Supplementary file 1 [file sensors-22-09594-s001.zip › sensors-2058321-supplementary.pdf]

Supplementary Materials

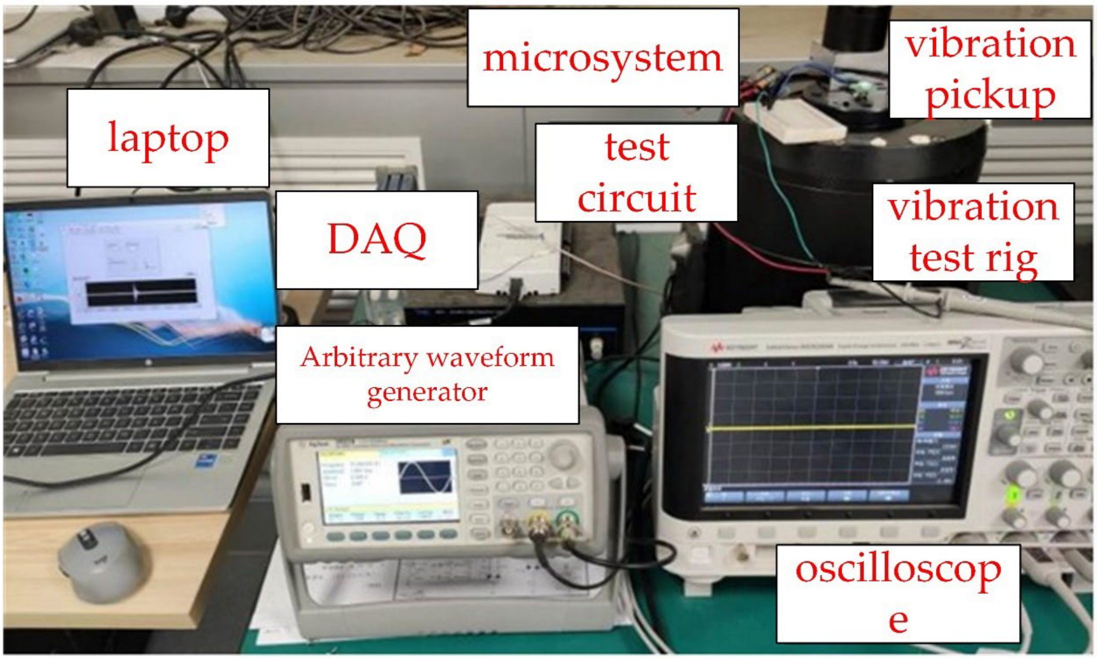

Supplementary Figure S1. Indoor experiment setup.

Supplementary Table S1. Comparison of signal operation computation.

Stage	Operation	Main operations	Computation volume
pre-processing	mean	sum / divide	low
	median	sum / divide	low
	covariance	sum / product/ divide	lower
	empirical mode decomposition	derivative / interpolation	high
feature extraction	zero crossing number	difference / sum	low
	maximum adjacent zero-crossing time	difference / sum	low

	interval		
	peak frequency	Fourier transform	lower
	Mel Cepstrum Coefficient	derivative / interpolation / Fourier transform / autocorrelation	very high
	cepstral analysis	Fourier transform / logarithmic / inverse Fourier	High
classification	threshold	N/A	Low
	RNN	N/A	very high
	SVM	N/A	Low

Supplementary Table S2. SVM code.

```
import numpy as np
from sklearn import svm
from sklearn.metrics import accuracy_score
from sklearn.model_selection import train_test_split
from sklearn.preprocessing import StandardScaler
import pandas as pd
import matplotlib.pyplot as plt

def f_importances(coef, names):
    imp = coef
    imp,names1 = zip(*sorted(zip(imp, names)))
    plt.bar(range(len(names)), list(imp[0]), align='center')
    plt.xticks(range(len(names)), names)
    plt.show()

def main():
    data = pd.read_csv("./vehicle-tracker.csv")
    pd.set_option('display.max_columns', None)
    print(data.columns)
    print(data.head(5))
    print(data.describe())
    # 特征选择
    feature_names = ['sum', 'crosszeros1', 'crz1time', 'hz',
'crz2time']
```

```python
    train_X = data[['sum', 'crosszeros1', 'crz1time', 'hz',
'crz2time']].values
    train_y = data[['leibie']].values.ravel()
    # Take 30% of the data as the test set and the rest as the
training set
    X_train, X_test, y_train, y_test = train_test_split(train_X,
train_y, test_size=0.3, random_state=101)
    # Extract the value of feature selection as training and test
data
    # Z-Core is used to normalize data to ensure that the average
value of each feature dimension is 0 and the variance is 1
    ss = StandardScaler()
    train_X = ss.fit_transform(train_X)
    # Create SVM classifier
    model = svm.SVC(kernel='poly', gamma='auto', coef0=1.1)
    # Training Set
    model.fit(train_X, train_y)

    # ranking of features' importance
    supportShape = model.support_vectors_.shape
    nbSupportVectors = supportShape[0]
    vectorDimensions = supportShape[1]
    print("nbSupportVectors = %d" % nbSupportVectors)
    print("vectorDimensions = %d" % vectorDimensions)
    print("degree = %d" % model.degree)
    print("coef0 = %f" % model.coef0)
    print("gamma = %f" % model._gamma)
    print("intercept = %f" % model.intercept_)
    VECDIM = 5 # dimension 5
    dualCoefs = model.dual_coef_
    dualCoefs = dualCoefs.reshape(nbSupportVectors)
    print("Dual Coefs")
    print(dualCoefs)
    supportVectors = model.support_vectors_
    supportVectors = supportVectors.reshape(nbSupportVectors *
VECDIM)
    print("Support Vectors")
    print(supportVectors)
    np.savetxt('human-vehicle—dualCoefs-poly.csv', dualCoefs,
delimiter=',')
    np.savetxt('human-vehicle-poly.csv', supportVectors,
delimiter=',')
    # Use test set for prediction
    prediction = model.predict(X_test)
```

```
    print('poly kernel accuracy: ', accuracy_score(y_test,
prediction))
    # Create SVM classifier
    model = svm.SVC(gamma=0.001, C=100., kernel = 'linear')
    # training sets
    model.fit(train_X, train_y)
    # Use test set for prediction
    prediction = model.predict(X_test)
    print('linear kernel accuracy: ', accuracy_score(y_test,
prediction))
    # ranking of features' importance
    f_importances(model.coef_, feature_names)

if __name__ == '__main__':
    main()
```
